# Effect of brainwave entrainment on sleep quality among MBBS students in India

**DOI:** 10.6026/973206300220122

**Published:** 2026-01-31

**Authors:** Varun Malhotra, Abhay Gupta, Danish Javed, Francisco Jose Cidral-Filho, Patrick K Porter

**Affiliations:** 1 Department of Physiology, All India Institute of Medical Science (AIIMS), Bhopal, India; 2 Department of AYUSH, All India Institute of Medical Science (AIIMS), Bhopal, Madhya Pradesh, India; 3 Department of Health Sciences, Laboratory of Experimental Neurosciences (LaNEx), University of South Santa Catarina, Palhoca, Brazil; 4 Integrative Wellbeing Institute, Windermere, Florida, USA; 5 Research Laboratory of Posturology and Neuromodulation (RELPON), Department of Human Neuroscience, Sapienza University, Rome, Italy; 6 Department of Neuro-Linguistic Programming and Hypnotherapy, Quantum University, Honolulu, HI, USA; 7 Department of Mind Based Studies, BrainTap INC, New Bern, North Carolina, USA

**Keywords:** Brainwave entrainment, sleep architecture, auditory stimulation, sleep quality

## Abstract

Brainwave entrainment (BWE) synchronizes neural oscillations using rhythmic auditory and photic stimuli. Therefore, it is of interest to study the immediate effect of a single delta wave BWE session using the Brain Tap headset on sleep parameters among medical students. Hence, twenty six participants underwent one BWE session, with sleep metrics recorded pre and post session using a smart watch. Delta wave BWE was associated with significant improvements in sleep score, total sleep duration, light sleep and deep sleep, with reduced nocturnal awakenings. Thus, we show delta frequency BWE may serve as a non-pharmacological aid for enhancing sleep quality.

## Background:

Sleep is essential for physical and mental health, supporting cardiovascular regulation, cognition, memory, immunity, reproductive and hormonal balance, while insufficient or disrupted sleep arising from conditions such as insomnia, sleep apnea, circadian disturbances, or lifestyle factors contributes to significant morbidity [1]. Sleep maintains immune homeostasis through its bidirectional interaction with the immune system, in which immune activation alters sleep patterns while chronic sleep deficiency induces low-grade systemic inflammation that increases the risk of diabetes, atherosclerosis and neurodegenerative disorders, underscoring its vital role in physiological resilience [2]. Sleep disturbances are highly prevalent among university students, who often experience irregular schedules, short sleep duration and poor sleep quality due to maturational, psychosocial and academic demands, with evidence showing that inadequate sleep manifesting as daytime drowsiness, sleep deprivation, or irregular sleep wake patterns negatively affects academic performance [3]. University students experience high psychological stress that disrupts sleep and increases insomnia, while poor sleep further heightens stress and emotional dysregulation, together contributing to reduced well-being and impaired academic performance [4]. Medical students experience substantial sleep disruption due to the demands of their training, with nearly 40% reporting poor sleep quality, about 29% insufficient sleep duration and over 35% excessive daytime sleepiness and since academic performance correlates more strongly with sleep quality and continuity than with total duration, targeted interventions to improve sleep quality are essential for supporting their learning and overall health [5]. Given the strong links between sleep, immunity and cognitive performance and the high prevalence of sleep problems in university and medical students, there is a growing need for accessible non-pharmacological interventions such as brainwave entrainment, which uses rhythmic auditory and photic stimuli delivered through audiovisual, auditory, or photic methods to synchronize neural oscillations via the frequency-following response and promote relaxation, focus, or sleep by driving brain activity toward targeted frequencies [6]. Previous studies have reported a range of physiological and psychological benefits associated with brainwave entrainment (BWE). BWE has been shown to improve subjective ratings of sleep and awakening quality, reduce sleepiness and enhance motivation in young elite athletes; decrease migraine frequency; enhance attention; reduce anxiety; reduce confusion and fatigue while increasing vigor and activity; and improve heart rate variability (HRV), a marker of autonomic balance and relaxation [7, 8, 9, 10, 11, 12-13]. Therefore, it is of interest to assess the effect of single delta-wave brainwave entrainment session delivered through the BrainTap® headset for immediate, objectively measurable improvements in sleep quantity and quality among medical students by targeting delta frequencies associated with deep, restorative sleep.

## Methods:

## Study design:

This study employed a single-group pretest-posttest (within-subject) design to evaluate the immediate effects of a delta wave audio- based BWE session on sleep parameters. Participants were undergraduate students enrolled in the Bachelor of Medicine, Bachelor of Surgery (MBBS) program at the All India Institute of Medical Sciences (AIIMS), Bhopal. Students who expressed interest were invited to participate and were required to provide written informed consent and complete a screening questionnaire prior to enrollment. The study was conducted in accordance with the ethical principles of the Declaration of Helsinki and the research protocol received approval from the AIIMS Institutional Human Ethics Committee (approval number: LOP IM0425).

## Inclusion and Exclusion criteria:

Inclusion criteria comprised male or female participants aged 18 years or older, with proficiency in English and willingness to voluntarily participate in the study. Exclusion criteria included previous use of brainwave entrainment devices or applications, hearing impairments, serious physical illness, chronic diseases, or significant health complications, use of prescribed sleep or anxiety medications, pregnancy and any diagnosis of sleep disorders or psychiatric illness, as well as ongoing psychotropic medication use.

## Procedures:

Delta wave audio-based brain wave entrainment (BWE) sessions were delivered using the BrainTap® headset and application. Each session integrated background music, guided meditation and synchronized brainwave entrainment through binaural beats and isochronic tones, combined with photic stimulation provided by the headset's light- emitting diodes. This multimodal approach was designed to promote relaxation and enhance delta wave activity. Sleep-related parameters were recorded before and after the BWE session using the Amazfit Active Edge 46 mm Smart watch (AI Health Coach for gym, outdoor activity and exercise; ultra-long 16-day battery; 10 ATM water resistance; Android/iOS compatible; GPS-enabled, Mint Green model). Participants wore the smart watch during sleep and the device automatically tracked sleep architecture and related physiological metrics for subsequent analysis.

## Statistical analysis:

Data were analyzed using paired-sample t-tests to compare pre- and post- session sleep parameters obtained from the Amazfit smart watch. Prior to analysis, data were screened for outliers and assess for normality using the Shapiro-Wilk test. For variables in which the assumption of equal variances was violated according to Levene's test (p < .05), the corresponding adjusted t-test values were reported. Results are expressed as mean ± standard deviation (SD). For each variable, the mean difference, standard error of the difference, 95%confidence interval (CI) and effect size (Cohen's d) were calculated to estimate the magnitude of change. All values were computed using the formula baseline - post. Negative mean differences and corresponding t/d values indicate that post-session scores were numerically higher than baseline values. Statistical significance was set at p < 0.05 (two-tailed). All statistical analyses were performed using Jamovi software (version 2.6.44).

## Results and Discussion:

**Figure 1:**
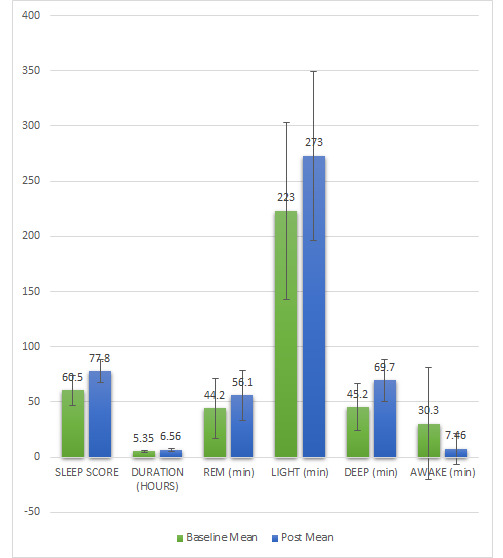
Mean and median changes in sleep parameters before and after the BWE

**Figure 2:**
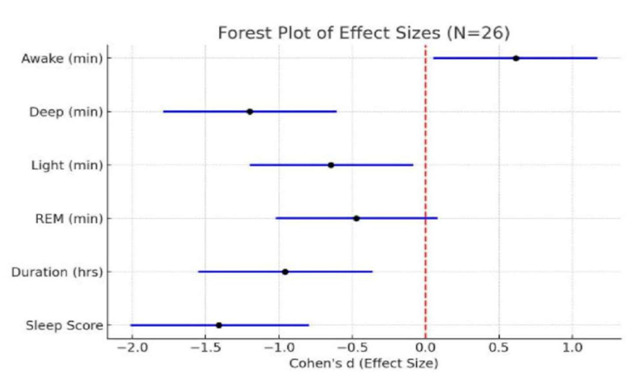
Forest plot of effect sizes for sleep parameters following the BWE Session

A total of twenty-six students (21) male, (5) female, (20years) mean age) participated in the study. Descriptive statistics for all sleep parameters are presented in Table 1. As illustrated in Figure 1, Figure 2 which display the distribution of mean and median values before and after the intervention, analysis revealed significant improvements in multiple sleep parameters following the delta wave brainwave entrainment (BWE) session. The sleep score increased markedly (p < .001,d = - 1.41), accompanied by significant gains in total sleep duration (p = .002, d = - 0.96), light sleep (p = .024, d = - 0.65) and deep sleep (p < .001, d = - 1.20). Conversely, time spent awake during the night significantly decreased (p = .032, d = 0.61). Although REM sleep exhibited an upward trend, the change did not reach statistical significance (p = .095). Collectively, these findings indicate that a session of delta wave BWE was associated with meaningful improvements in both the quality and quantity of sleep among participants. This study investigated the immediate effects of a single delta wave brainwave entrainment (BWE) session using the BrainTap headset on sleep parameters among medical students. Significant improvements were observed in sleep score, total sleep duration, light sleep and deep sleep, accompanied by a reduction in nocturnal awakenings. These results indicate that a short delta wave BWE session may acutely enhance restorative aspects of sleep architecture in healthy young adults. The increase in total sleep time and deep sleep duration suggests an enhancement of slow-wave sleep (SWS), the stage linked with physical recovery and memory consolidation [14, 15]. Although REM sleep also increased, the change did not reach statistical significance, possibly due to inter- individual variability or the short observation period. The reduction in time awake during the night further supports a potential improvement in sleep continuity and stability.

Delta oscillations, which dominate slow-wave sleep and arise from thalamocortical circuits that regulate non-REM sleep depth and continuity, play a central role in neural synchronization and restorative function; thus, the deeper sleep observed in this study may reflect a transient enhancement of thalamocortical synchronization-consistent with experimental findings showing that reduced delta activity characterizes insomnia while increased delta power indicates improved restorative quality-even though these mechanisms were not directly measured here [16]. Coordinated delta activity also extends beyond the cortex: cerebello- hippocampal recordings have shown delta-frequency coherence during non-REM sleep, supporting offline communication between these regions [17]. This network-level synchronization underscores the integrative role of delta oscillations in sleep-related restoration and could help explain the observed increase in deep sleep duration. Furthermore, population studies demonstrate that reduced delta wave entropy during sleep predicts higher long-term risks of cardiovascular disease and mortality, reinforcing the physiological relevance of delta activity as an indicator of systemic recovery [18]. From a mechanistic perspective, rhythmic auditory cues may promote these effects through the frequency-following response (FFR), a scalp-recorded potential produced by synchronized neural firing along the auditory pathway [19]. This mechanism explains how auditory entrainment can align brain oscillations with external rhythms. The addition of synchronized photic stimulation may further strengthen sensory coherence and enhance the entrainment effect. Evidence from binaural-beat research further supports these interpretations. Fan *et al.* demonstrated that 0.25 Hz binaural beats shortened the latency to slow-wave sleep during naps, suggesting that low-frequency rhythmic stimulation can prime thalamocortical networks for SWS [20]. Similarly, Dabiri *et al.* exposed participants to 90-minute delta binaural beat sessions each night for one week, following a baseline week without stimulation [21]. Using sleep diaries and the Profile of Mood States (POMS), they reported significant improvements in sleep duration; sleep quality, number of awakenings and post-wake mood, along with reductions in anxiety and anger. Although that study relied on audio-only stimulation and subjective assessments, while the present trial employed audiovisual entrainment and objective smartwatch-based monitoring, both sets of findings converge in demonstrating that delta-frequency stimulation can acutely and cumulatively enhance restorative sleep and emotional state. Beyond the electrophysiological perspective, deep sleep contributes to cellular recovery, hormonal balance and immune regulation, while REM sleep supports emotional regulation and overall sleep continuity [22, 23]. The observed improvements in sleep duration and reduced awakenings therefore indicate an acute enhancement of sleep stability and restorative depth, consistent with healthy sleep patterns. These results are particularly relevant for university and medical students, a population frequently exposed to stress, irregular schedules and sleep deprivation that compromise academic performance and well-being. Short, technology-assisted interventions such as BWE may offer a practical, non-pharmacological strategy to mitigate these challenges by improving sleep quality and recovery. However, this exploratory study has limitations. The single group pretest posttest design precludes causal inference and sleep metrics were obtained from a consumer-grade wearable rather than polysomnography. The modest, homogeneous sample further limits generalizability and only acute effects were evaluated. Future randomized controlled studies incorporating EEG- derived delta power and heart-rate variability should clarify the underlying neural and autonomic mechanisms and determine whether repeated sessions yield cumulative benefits.

## Other therapeutic uses of brain entrainment:

Brainwave entrainment is highlighted as a promising non-invasive therapeutic approach for neurological and psychiatric disorders in this 2024 review. The core concept is "oscillopathy"-the disruption of normal brainwave patterns. The paper explores techniques like Non-Invasive Brain Stimulation (NIBS), Neurologic Music Therapy (NMT), gamma stimulation, and somatosensory interventions using light or sound to correct these dysfunctional oscillations and potentially delay disease progression [24]. Auditory binaural beats and visual stimuli were found to significantly reduce the required dose of the sedative propofol in children undergoing surgery with regional anesthesia, according to this 2020 randomized controlled trial. The brainwave entrainment group required a mean infusion rate of 3.0 mg/kg/h compared to 4.2 mg/kg/h in the control group, with similar sedation levels achieved [25]. A novel method for automatically detecting and classifying brainwave entrainment beats (e.g., for alpha or beta waves) within music audio files is proposed in this 2023 computer science article. Using deep learning models (VGGish and YAMNET), the method achieved high accuracy (over 94%), offering an efficient alternative to EEG-based classification for music therapy and content moderation [26]. Two interventions for teenagers' mental health were assessed: a 4-week Heartfulness Meditation program significantly improved mood, stress, and anger, while audio brainwave entrainment alone did not show significant benefits. However, combining entrainment with meditation potentially enhanced sleep quality and further reduced stress compared to meditation alone [27]. Brainwave entrainment, delivered via a "David delight plus device," proved to be an effective non-pharmacological intervention for significantly reducing pre-operative fear and anxiety in children (aged 7-12) undergoing dental treatment, as demonstrated by this 2024 randomized controlled trial involving 252 participants [28]. Visual brainwave entrainment's effects on children with and without ADHD were explored in this 2024 study. Changes in resting-state brainwaves post-entrainment correlated with cognitive performance. A lower theta- beta ratio (TBR) was associated with better selective attention and working memory, particularly in children with ADHD, suggesting TBR as a key marker and entrainment as a potential therapeutic tool [29]. Regarding the use of brainwave entrainment for chronic pain, this is an editorial comment or correspondence piece that cites a 2020 study on the acceptability of smartphone-based entrainment for chronic pain [30]. Integrating music therapy, brainwave entrainment (especially gamma frequency), and AI-driven biofeedback to create adaptive, real-time interventions could optimize outcomes for mental health and cognitive rehabilitation, proposes this 2025 review on the future framework for personalized digital therapeutics [31]. Participant interviews revealed that a 4-week pre-sleep audio or visual alpha entrainment program was acceptable and feasible for home use by people with chronic pain and sleep disturbance. Users reported perceived benefits for both sleep and pain symptoms, with comfort and choice of stimulation type being important factors [32]. A new ultrasound neuromodulation technique called theta burst ultrasound stimulation (TBUS) was introduced in this 2024 preclinical mouse study. TBUS successfully induced long-lasting, bidirectional changes in brain plasticity in the motor cortex and enhanced motor skill learning, pointing to its potential as a powerful non-invasive tool for modulating brain function [33]. Binaural beat brainwave entrainment was investigated as a treatment for tinnitus in individuals with normal hearing. This 2025 study found the intervention to be a potentially effective therapeutic option for reducing tinnitus distress and its associated symptoms in this specific patient group [34]. Examining the association between different fear and anxiety scales when using brainwave entrainment in pediatric dentistry, this 2024 randomized controlled trial concluded the intervention was effective and established a strong positive correlation between the Visual Facial Anxiety Scale (VFAS) and Frankl's Behavior Rating Scale (FBRS) for measuring preoperative anxiety in children [35].

A virtual reality (VR)-based audio-visual brainwave entrainment system was explored to enhance learning and cognitive functions in children with ADHD. The 2025 study's results indicated that this immersive, multi-sensory entrainment approach could be a promising non- pharmacological tool for improving attention and learning outcomes [36]. Evaluating real-world application, this 2020 study found a smartphone-based brainwave entrainment application to be highly acceptable and easy to use for individuals managing chronic pain at home. Participants reported perceived benefits for pain, sleep, and relaxation, supporting its feasibility as a home-based therapeutic tool [37]. In individuals with normal hearing and bothersome tinnitus, a 30-day regimen of delta frequency binaural beats showed greater reductions in tinnitus handicap, depression, and anxiety compared to a control group receiving white noise. This 2024 study concludes delta wave binaural beats are a promising therapeutic tool, warranting further research despite limited quality-of-life improvements [38]. A rapid, objective method to assess alertness was developed using brief flickering light to induce brainwave entrainment while recording EEG. This 2024 study found specific EEG features during entrainment correlated with alertness states, and a machine learning model could classify alertness with high accuracy (AUC=0.90) without altering the subject's state [39]. Extending previous research, this 2025 study evaluated binaural beat treatment for tinnitus over three months, comparing delta and alpha beats to a standard masker. All groups improved, but binaural beats showed superior benefits in reducing distress and improving quality of life, strongly supporting their use as an effective, long-term treatment option [40]. Neurologic Music Therapy (NMT) and its impact on various neurological disorders are the focus of this 2024 review. It discusses how structured musical and rhythmic interventions can address cognitive, sensory, and motor dysfunctions resulting from neurological illness [41]. Exploring the role of gamma (γ) brain oscillations, this 2025 review delves into how modulating these high-frequency brainwaves is linked to cognitive functions and how their dysregulation serves as a potential biomarker for disorders like Alzheimer's and schizophrenia, positioning gamma neuromodulation as both a diagnostic tool and therapeutic target [42].

## Conclusion:

We show that a single delta waves BWE session using the BrainTap headset is associated with significant short-term improvements in multiple sleep parameters. Data show the feasibility and potential value of BWE as a simple, technology-assisted approach to promote sleep quality in healthy adults. Further controlled investigations are needed to confirm these findings and clarify the mechanisms involved.

## Funding:

Nil

## Figures and Tables

**Table 1:** Changes seen in different parameters from baseline to intervention

**Parameter**	**Mean ± SD Baseline**	**Mean ± SD Post**	**Student's t Statistic**	**p**	**Mean difference**	**SE difference**	**Effect Size Cohen's d**	**95% Confidence Interval**
SLEEP SCORE	60.5±13.8	77.8±10.4	-5.08	<.001	-17.2	3.39	-1.41	-2.01 to -0.793
DURATION (HOURS)	5.35±0.801	6.56±1.58	-3.35^a^	0.002	-1.21	0.36	-0.958	-1.55 to -0.361
REM (min)	44.2±27.5	56.1±22.8	-1.7	0.095	-11.9	7	-0.473	-1.02 to 0.0812
LIGHT (min)	223±79.8	273±76.7	-2.33	0.024	-50.5	21.7	-0.645	-1.20 to -0.0840
DEEP (min)	45.2±21.4	69.7±19.2	-4.34	<.001	-24.5	5.64	-1.2	-1.79 to -0.607
AWAKE (min)	30.3±50.8	7.46±13.8	2.21^a^	0.032	22.8	10.3	0.614	0.0539 to 1.17
N=26, dF=50, Note.
H_a_ µBaseline ≠ µPost
^a^ Levene's test is significant (p < .05),
suggesting a violation of the assumption of equal variances
